# Efficacy and Limitations of rCBF-SPECT in the Diagnosis of Alzheimer’s Disease With Amyloid-PET

**DOI:** 10.1177/1533317519841192

**Published:** 2019-04-09

**Authors:** Miwako Takahashi, Tomoko Tada, Tomomi Nakamura, Keitaro Koyama, Toshimitsu Momose

**Affiliations:** 1 Division of Nuclear Medicine, Department of Radiology, Graduate School of Medicine, The University of Tokyo, Tokyo, Japan; 2 Eli Lilly Japan K.K., Kobe, Japan

**Keywords:** regional cerebral blood flow SPECT, amyloid-PET, Alzheimer’s disease, mild cognitive impairment, florbetapir-PET

## Abstract

This study aimed to assess efficacy and limitations of regional cerebral blood flow imaging using single-photon emission computed tomography (rCBF-SPECT) in the diagnosis of Alzheimer’s disease (AD) with amyloid-positron emission tomography (amyloid-PET). Thirteen patients, who underwent both rCBF-SPECT and amyloid-PET after clinical diagnosis of AD or mild cognitive impairment, were retrospectively identified. The rCBF-SPECTs were classified into 4 grades, from typical AD pattern to no AD pattern of hypoperfusion; amyloid-beta (Aβ) positivity was assessed by amyloid-PET. Four patients were categorized into a typical AD pattern on rCBF-SPECT, and all were Aβ+. The other 9 patients did not exhibit a typical AD pattern; however, 4 were Aβ+. The Mini-Mental State Examination score and Clinical Dementia Rating scale were not significantly different between Aβ+ and Aβ– patients. A typical AD pattern on rCBF-SPECT can reflect Aβ+; however, if not, rCBF-SPECT has a limitation to predict amyloid pathology.

## Introduction

Rapid increases in aging populations are associated with increases in the number of patients with complaints of cognitive impairment.^1,2^ Japan has the most elderly population among developed countries and is currently experiencing difficulties in treating and caring for these patients.^3,4^ The most prevalent cause of cognitive impairment in the elderly individuals is Alzheimer’s disease (AD). Amyloid-positron emission tomography (amyloid-PET) can detect amyloid pathology with high sensitivity and specificity,^5^ and it helps to diagnose AD.

Measurement of regional cerebral blood flow (rCBF) using single-photon emission computed tomography (rCBF-SPECT) is important not only for better understanding of patient symptoms but also for determining underlying pathophysiologies. The rCBF reflects neuronal synaptic activity by virtue of autoregulation, which consumes most of the energy delivered by glucose. Therefore, rCBF-SPECT enables the evaluation of regional decreases in neuronal activity. Decreased activity in specific areas and the severity of such provide insight into the underlying pathology of the cognitive impairment. In many cases of AD, typical patterns of hypoperfusion are found on rCBF-SPECT and deteriorate longitudinally.

Typical AD patterns of rCBF may be helpful and are widely used for the diagnosis of dementia in clinical settings.^6-14^ However, not all patients with suspected AD exhibit typical AD patterns because the distribution of rCBF varies widely, even in patients with the same underlying AD pathology due to small infarctions, vascular stenosis, and other degenerative conditions, such as widespread tau pathology. In contrast, amyloid-PET imaging reflects the density of amyloid plaque, with less influence from other potential comorbidities. More specifically, low levels of amyloid revealed in a PET scan suggest the absence or sparse neuritic plaques and are inconsistent with a diagnosis of AD and vice versa.

Only a few works have been reported on a comparison between SPECT and amyloid-PET, but they still have limitations such as few participants and unified SPECT tracers.^15,16^ In this study, we addressed the predictive value of typical hypoperfusion patterns on rCBF-SPECT for AD pathology and attempted to evaluate the efficacy and limitations of rCBF-SPECT in the diagnosis of AD based on the results of the amyloid-PET in a clinical setting. Amyloid pathology was confirmed by amyloid-PET using florbetapir.^17, 18^ We also evaluated dementia severity using the Mini-Mental State Examination (MMSE) and Clinical Dementia Rating (CDR) scale.

## Methods

### Study Population

We retrospectively identified the consecutive patients who underwent rCBF-SPECT in clinical setting prior to florbetapir PET which is performed as part of a phase 2/3 clinical trial (AVBB, ClinicalTrials.gov Identifier: NCT01662882) for florbetapir and were selected for this study.^19^ A total of 48 patients originally participated in AVBB study including 15 patients with AD, 15 patients with MCI, and 18 controls. Among those patients, 13 patients underwent rCBF-SPECT and were analyzed for this study.

Written informed consent was obtained from all patients, and the study protocol was approved by the institutional review board of the hospital. We carefully checked magnetic resonance imaging (MRI) of each patient to exclude possible other causes that would have effect on cognitive impairment or rCBF-SPECT. We excluded patients who had moderate or severe abnormalities on MRI such as multiple infarction, diffuse atrophy, localized severe atrophy extended over 1 gyrus, or diffuse hyperintensity on T2-weighted imaging, but we included the patients with slight atrophy of hippocampal area. The MRI findings at screening of 2 or more infarcts or lacunes or severe white matter disease were also exclusionary.^19^ There was no patients excluded based on MRI findings.

All patients were clinically diagnosed with AD or mild cognitive impairment (MCI) based on consensus clinical diagnostic criteria.^20,21^ Nine patients (1 man and 8 women; mean age 76 ± 4 years) were clinically diagnosed with AD, and 4 (2 men and 2 women; mean age 78 ± 5 years) were diagnosed with MCI. The rCBF-SPECT was performed earlier than amyloid-PET by an average of 13.7 months (range: 4-47 months). Magnetic resonance imaging, MMSE, and CDR were performed at the time of amyloid-PET. Details regarding MMSE and CDR global scores of the patients are summarized in Table 1.

**Table 1.** Patient Profiles and Results of CBF-SPECT and Amyloid-PET.^a^

Case	Age	Sex	Clinical Diagnosis	MMSE	CDR	CBF-SPECT	Amyloid-PET	Interval^b^, months
1	71	M	AD	23	1	Likely	Aβ+	4
2	79	F	AD	23	1	Unlikely	Aβ+	12
3	76	F	AD	17	1	Likely	Aβ+	11
4	71	F	MCI	26	0.5	Very likely	Aβ+	12
5	75	F	AD	21	1	Unlikely	Aβ+	24
6	77	F	AD	13	1	Very likely	Aβ+	13
7	77	M	MCI	27	0.5	Very likely	Aβ+	4
8	67	F	AD	23	0.5	Very likely	Aβ+	7
9	78	F	AD	18	2	Likely	Aβ–	47
10	78	M	MCI	26	0.5	Likely	Aβ–	6
11	78	F	AD	24	0.5	Likely	Aβ–	19
12	80	F	AD	24	1	Unlikely	Aβ–	9
13	84	F	MCI	25	0.5	Unlikely	Aβ–	10

Abbreviations: AD, Alzheimer’s disease; Aβ, amyloid β; Amyloid-PET, amyloid positron emission tomography; CBF-SPECT, cerebral blood flow imaging using single-photon emission computed tomography; CDR, Clinical Dementia Rating; MMSE, Mini-Mental State Examination; M, male; F, female; MCI, mild cognitive impairment.

^a^Cases are re-sorted according to Aβ+ (case 1 to 8) or Aβ– (case 9 to 13).

^b^Interval of regional cerebral blood flow (rCBF) using single-photon emission computed tomography (rCBF-SPECT) and amyloid-positron emission tomography (PET). In all patients, rCBF-SPECT scans were performed earlier than amyloid-PET.

### rCBF-SPECT

Commercially available I-123-labeled iodoamphetamine (IMP; Nihon Medi-Physics, Nishinomiya, Japan) was used for rCBF-SPECT scanning. Patients were rested in the supine position with an eye mask in a quiet SPECT room to minimize confounding factors of environmental noise. A 222-MBq (6 mCi) dose of IMP was injected intravenously and, 35 minutes later, a 30-minute scan was performed using a triple-head SPECT system (GCA-9300R; Toshiba Medical System, Otawara, Japan) equipped with a high-resolution fan-beam collimator, which permitted a spatial resolution of 7.2 mm full-width at half-maximum (FWHM). Neither photon attenuation correction nor scatter correction was performed. The SPECT images were reconstructed using filtered back projection with Butterworth and Ramp filter. The data were collected in a 128 × 128 × 89 matrix with a voxel size of 1.72 × 1.72 × 3.44 mm.

### Amyloid-PET

Florbetapir was prepared as described previously.^22^ The doses of florbetapir were manufactured as an investigational medical product based on clinical good manufacturing practices in accordance with Avid Radiopharmaceuticals Investigational New Drug master batch record and quality control release criteria (Avid Pharmaceuticals, Philadelphia, Pennsylvania). A 370-MBq (10 mCi) dose of florbetapir was injected intravenously, and a 10-minute emission PET/computed tomography (CT) scan (Discovery 690; GE Medical Systems, Milwaukee, Wisconsin) was performed, beginning 50 minutes after injection. The intrinsic FWHM spatial resolution was 4.9 mm. The PET images were reconstructed using a Fourier rebinning algorithm with ordered subset expectation maximization iterative reconstruction, with 2 iterations and 8 subsets and a 4-mm FWHM Gaussian filter. The data were collected in a 256 × 256 × 47 matrix with a voxel size of 2.0 × 2.0 × 3.27 mm.

### Image Assessment

The rCBF-SPECT images were anonymized, then reevaluated visually by 3 nuclear medicine experts (MT, KK, and TM) independently without clinical information and the results of amyloid-PET, and categorized into 1 of 4 grades as follows:

- very likely (hypoperfusion area is a typical AD pattern);
- likely (hypoperfusion area closely resembles an AD pattern);
- unlikely (hypoperfusion area is partially shared with an AD pattern); and
- very unlikely (hypoperfusion area is not an AD pattern)

The categorization was based on previous agreement among raters that a typical AD pattern on rCBF-SPECT demonstrated selective hypoperfusion in the temporoparietal area, posterior cingulate, and precuneus and hippocampal areas in which the side of the greater deficit was consistent across these regions, and the primary sensorimotor areas, cerebellum, basal ganglia, and thalamus were relatively preserved.^5-7,10-13^ The degree of hypoperfusion was judged comparing the reference areas that assumed to be normal cortex. If the evaluated area, such as temporoparietarl area, posterior cingulate, precuneus, hippocampal area, primary sensorimotor area, cerebellum, basal ganglia, and thalamus, shows yellow or lower color than the assumed normal area showing orange to red on the colorbar, we judged them as hypoperfusion. We allowed the hypoperfusion of the frontal lobe or occipital lobe if the degree was mild and less severe to the specific area of AD pattern. The visual evaluation used axial view and coronal view images.

**Figure 1.**
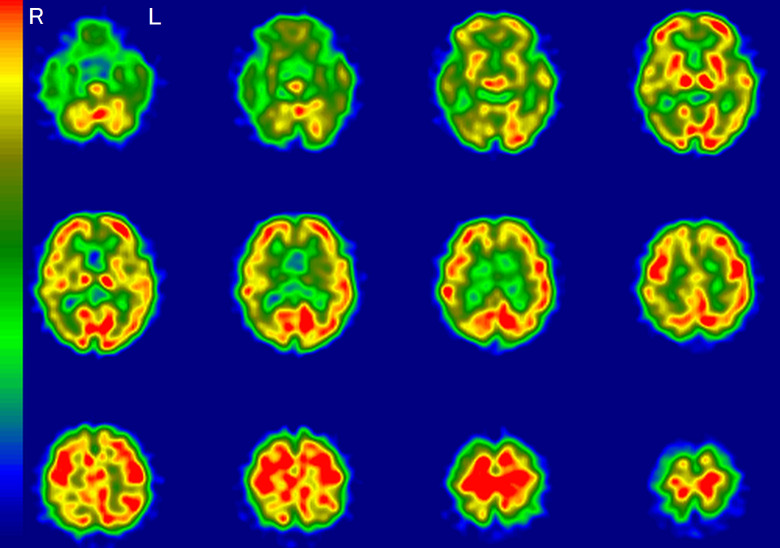
A typical Alzheimer’s disease pattern on regional cerebral blood flow imaging using single-photon emission computed tomography (rCBF-SPECT). Hypoperfusion is apparent in the temporoparietal, hippocampal, posterior cingulate, and precuneus regions. The right (R) side is consistently more decreased throughout these areas. L indicates left.

A representative image of a typical AD pattern is shown in Figure 1. This typical AD pattern was defined as “very likely.” Accordingly, “likely” meant that hypoperfusion was found in the same area as the typical AD pattern, except for 1 or 2 areas. “Unlikely” meant that hypoperfusion was found in no more than 1 or 2 areas of the typical AD patterns. “Very unlikely” was defined as the absence of hypoperfusion in the typical AD areas. If there was disagreement with regard to classification among the raters, the majority rating was adopted.

Amyloid-PET images were visually classified as amyloid β positive (Aβ+) or amyloid β negative (Aβ–) by 5 readers (United States-trained nuclear medicine physicians) according to previously published criteria.^5^ Readers were blinded to all subject information including rCBF-SPECT data. If there was a disagreement among the raters, the majority classification was adopted.

## Results

All 3 raters completely agreed in the categorization of rCBF-SPECT in 4 patients (case 2, 5, 9 and 12). For the other 9 patients, raters judged differently although at least 2 of them accorded in the categorization. In such cases, we determined the classification upon which was categorized by those 2 raters. There were no cases in which all 3 raters categorized differently. Regarding the rating of florbetapir PET, since 5 raters categorize images as positive and negative, amyloid-PET positivity was determined by majority assessment.

**Figure 2.**
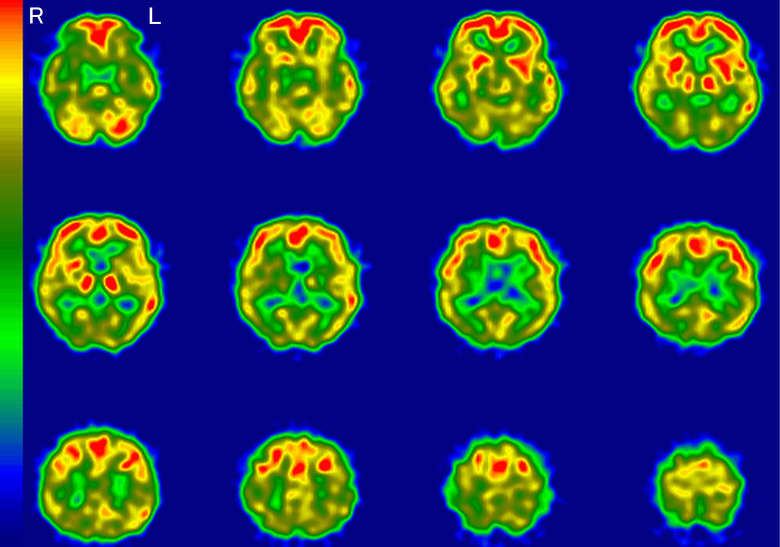
Regional cerebral blood flow imaging using single-photon emission computed tomography (rCBF-SPECT) images from case 4 who was clinically diagnosed with mild cognitive impairment (MCI) and is representative of an amyloid β (Aβ)+ patient with rCBF-SPECT graded as “very likely.” The rCBF is reduced in the temporoparietal, hippocampal, posterior cingulate, and precuneus areas. The right (R) side is consistently more decreased throughout these areas. L indicates left.

**Figure 3. fig3-1533317519841192:**
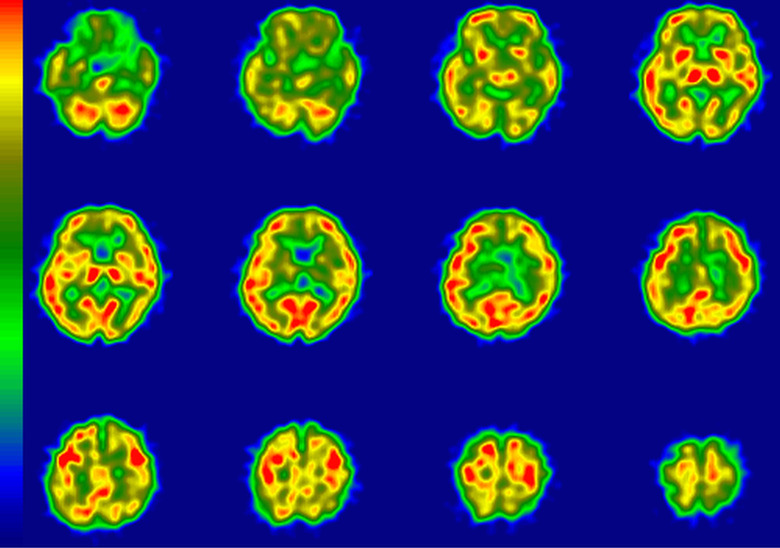
Regional cerebral blood flow imaging using single-photon emission computed tomography (rCBF-SPECT) images from case 3 who was clinically diagnosed with Alzheimer’s disease (AD) and is representative of an amyloid β (Aβ)+ patient with rCBF-SPECT graded as “likely.” The rCBF is reduced in the left temporoparietal and hippocampal areas. The posterior cingulate and precuneus areas are relatively preserved. R indicates right; L, left.

**Figure 4. fig4-1533317519841192:**
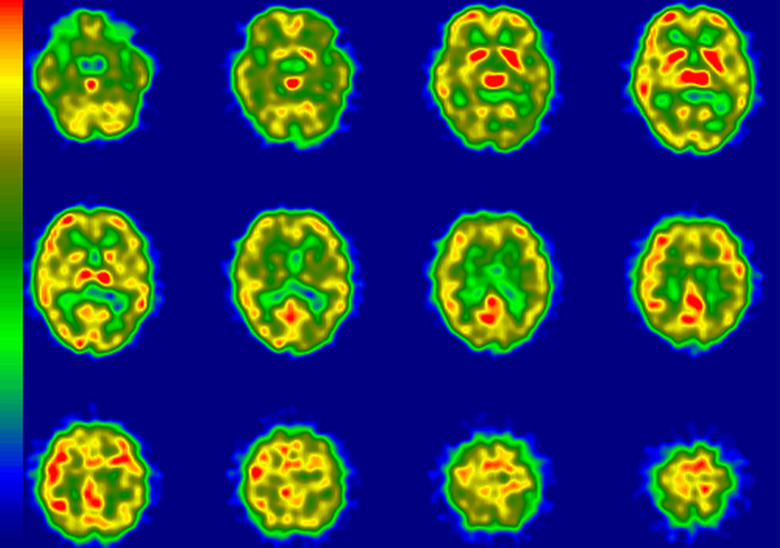
Regional cerebral blood flow imaging using single-photon emission computed tomography (rCBF-SPECT) images from case 9 who was clinically diagnosed with Alzheimer’s disease (AD) and is representative of an amyloid β (Aβ)– patient with rCBF-SPECT graded as “likely”. The rCBF is reduced dominantly in the left temporoparietal and bilateral hippocampal areas. The posterior cingulate and precuneus areas are relatively preserved. R indicates right; L, left.

The results of imaging assessment of rCBF-SPECT and amyloid positivity in amyloid-PET are summarized in Table 1. The Aβ+ patients were younger than their Aβ– counterparts (mean age 74.1 vs 79.6 years; *P* = .01 [*t* test]). The rCBF-SPECT images suggesting “very likely” were found in 4 patients, “likely” in 5, “unlikely” in 4, and no patients were identified with a rCBF pattern of “very unlikely.” Representative cases in rCBF-SPECT images are shown in Figures 2 [Fig fig3-1533317519841192] [Fig fig4-1533317519841192] to 5. All 4 patients with “very likely” were Aβ+. Two of 5 patients with “likely” and 2 of 4 with “unlikely” were Aβ+. No significant difference was apparent in the MMSE score among the 3 groups (“unlikely”, “likely,” and “very likely”) defined by rCBF-SPECT (Figure 6). In addition, there was no difference in MMSE score between Aβ+ and Aβ– patients (*P* = .43; Figure 6). Similarly, significant differences were not apparent in CDR global score among the 3 groups or between Aβ+ and Aβ– patients (*P* = .79; Figure 7).

**Figure 5.**
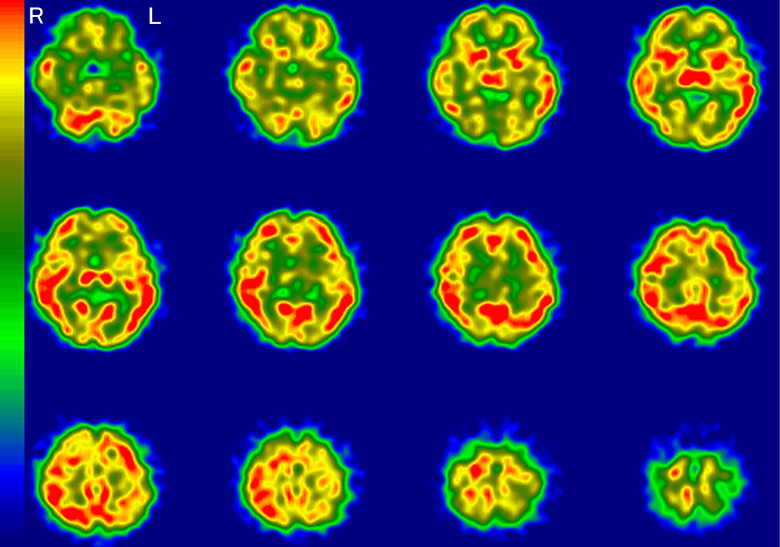
Regional cerebral blood flow imaging using single-photon emission computed tomography (rCBF-SPECT) images from case 5 who was clinically diagnosed with Alzheimer’s disease (AD) and is representative of an amyloid β (Aβ)+patient with rCBF-SPECT graded as “unlikely.” The rCBF is reduced in the hippocampal areas, dominantly in the left side. The bilateral temporoparietal areas are relatively preserved. R indicates right; L, left

**Figure 6.**
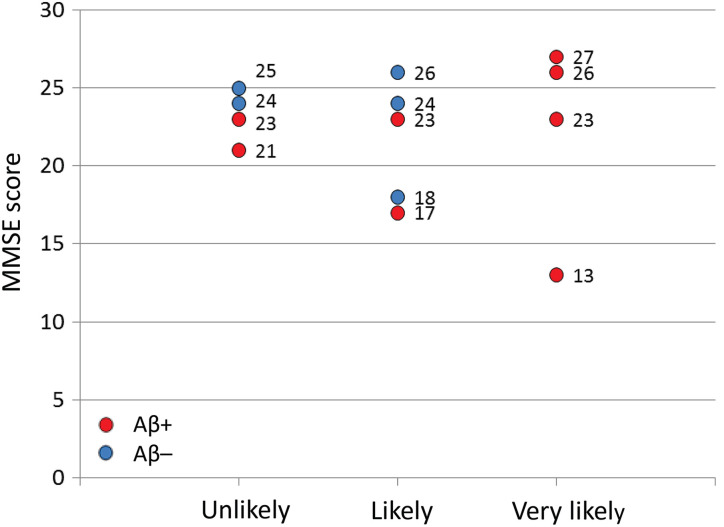
Mini-Mental State Examination (MMSE) scores in each patient according to regional cerebral blood flow (rCBF) grades. Red and blue circles represent amyloid β (Aβ)+ and Aβ–, respectively.

**Figure 7.**
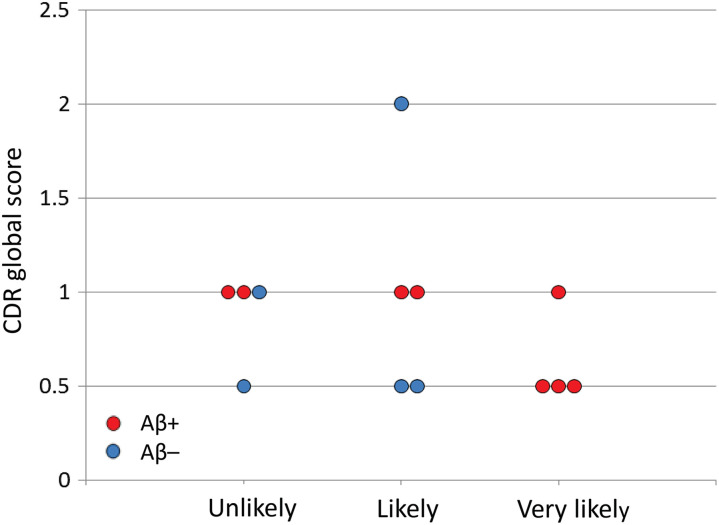
Clinical Dementia Rating (CDR) global scores in each patient according to regional cerebral blood flow (rCBF) grades. Red and blue circles represent amyloid β (Aβ)+ and Aβ–, respectively.

## Discussion

This was the study to compare the agreement of rCBF-SPECT and florbetapir PET in patients clinically diagnosed with MCI and AD. This study demonstrated that all 4 patients who exhibited a typical AD pattern of rCBF distribution were Aβ+ on amyloid-PET. This means that in patients with a clinical diagnosis of MCI or AD, a typical AD pattern of hypoperfusion on rCBF-SPECT suggests a high likelihood of AD pathology. In contrast, the absence of a typical AD rCBF pattern did not rule out the presence of AD pathology. Two of five patients with “likely” AD patterns on rCBF-SPECT and 2 of 4 with “unlikely” were Aβ+ in amyloid-PET. These results suggest that rCBF-SPECT imaging patterns are not always predictive of the amyloid status and, thus, may not reflect underlying AD pathology.

Compared to histopathological studies, temporoparietal decreases on rCBF-SPECT have provided a positive predictive value of 92% and negative predictive value of 57% for the diagnosis of AD in patients clinically diagnosed with possible dementia.^7^ Another study reported that a typical AD pattern on rCBF-SPECT increased the likelihood of AD to 92% when the clinical diagnosis of “probable” AD was associated with an 84% likelihood of pathological AD.^23^ This has also been confirmed in longitudinal clinical studies.^11^ Amyloid PET using a florbetapir tracer reflects the presence and extent of amyloid pathology at autopsy measured using immunohistochemistry.^17,18^ In the present study, we demonstrated that a typical AD rCBF-SPECT pattern had a high positive predictive value for AD pathology, whereas less typical patterns had relatively modest negative predictive value among patients with a clinical diagnosis of MCI or AD.

The present study revealed a limitation of rCBF-SPECT to predict amyloid status using pathophysiological indicators, such as blood flow. For one reason, the progression of AD begins with an accumulation of amyloid, followed by the spread of tau beyond the temporal lobe, cerebral impairments, and, subsequently, deficits in glucose metabolism and, presumably, changes in rCBF.^24,25^ Therefore, the timing of conducting rCBF-SPECT and amyloid-PET scans can affect the findings of these examinations in assessing AD diagnosis. In fact, in the present study, rCBF-SPECT was performed 4 to 47months earlier than amyloid-PET. It is, therefore, possible that rCBF had not yet decreased to a level that could be identified visually as an AD pattern due to the earlier timing of the rCBF-SPECT in Aβ+ paients without a “very likely” or “likely” AD rCBF pattern. On the other hand, we believe that the interval of 47 months between CBF-SPECT and amyloid-PET would not affect the result of amyloid-PET because if the patients have already developed memory or cognitive dysfunction due to AD, they would already show detectable Aβ on amyloid-PET.

Although we did not notice any difference depending on age in evaluating rCBF-SPECT, the frontal lobe may be affected by aging. The rCBF-PET using ^15^O-CO_2_ inhalation steady state method demonstrated that superofrontal gyrus was identified with weakly significant linear decreases with advancing age.^26^ The other study using ^15^O-H_2_O intravenous bolus injection method revealed that the mean cortical CBF, and rCBF of lateral temporal and lateral orbitofrontal were lower in the elderly group (older than 50 y-o) compared to young/midlife group, but the significance disappeared after partial volume effect (PVE) correction. In the basal ganglia, thalamus, and cerebellum, no significant relationship between age and CBF was observed.^27^ In this study, we carefully excluded the patients with moderate to severe atrophy which may cause PVE, and evaluated rCBF on the areas such as the temporoparietal, posterior cingulate, precuneus, and hippocampal areas, and the consistencies of their lateralities. Therefore, we believe that the aging affect may be slight, if any, on our rCBF-SPECT classifications.

The most desirable confirmation of amyloid pathology is through histopathological analysis and diagnosis. Recently, amyloid-PET findings have been used as a surrogate for the diagnosis of amyloid pathology. We should note that the Aβ+ status on amyloid-PET can be linked to the presence of at least 1 component of AD pathology and does not always provide confirmation of AD. In brief, other pathologies—apart from amyloid—such as tau and Lewy bodies were not identified by amyloid-PET, and neurodegenerative diseases related to those pathologies were not ruled out. Multiple pathologies can be suggested by heterogeneous rCBF-SPECT findings even when amyloid-PET reveals Aβ+.

We have other limitations in this study. The sample size was small. Although we demonstrated that all of the patients who were suspected with clinical AD or MCI with typical AD pattern on rCBF-PET were Aβ+ on amyloid PET, we could not exclude the possibility that the case which is opposite to this result may exist. The interval between the timings of rCBF-SPECT and amyloid PET varied (up to a maximum of 47 months). Considering the decreases in rCBF progresses if the patients have AD pathology, rCBF-SPECT which is performed earlier may be more underestimated.

## Conclusion

The rCBF-SPECT may be supportive in providing insight into underlying AD pathology by revealing a typical AD pattern. However, the absence of a typical AD rCBF pattern did not rule out the presence of AD pathology. These results suggest that a typical AD pattern in rCBF-SPECT is associated with the presence of amyloid, which is consistent with an AD diagnosis; however, atypical patterns cannot reduce diagnostic uncertainty.
